# Spatial attention in encoding letter combinations

**DOI:** 10.1038/s41598-021-03558-4

**Published:** 2021-12-17

**Authors:** Mahalakshmi Ramamurthy, Alex L. White, Clementine Chou, Jason D. Yeatman

**Affiliations:** 1 Developmental-Behavioral Pediatrics, School of Medicine and Graduate School of Education, Stanford University, Stanford, CA USA; 2 Department of Neuroscience and Behavior, Barnard College, New York, NY USA

**Keywords:** Human behaviour, Attention, Dyslexia, Reading, Psychology

## Abstract

Reading requires the correct identification of letters and letter positions within words. Selective attention is, therefore, required to select chunks of the text for sequential processing. Despite the extensive literature on visual attention, the well-known effects of spatial cues in simple perceptual tasks cannot inform us about the role of attention in a task as complex as reading. Here, we systematically manipulate spatial attention in a multi-letter processing task to understand the effects of spatial cues on letter encoding in typical adults. Overall, endogenous (voluntary) cue benefits were larger than exogenous (reflexive). We show that cue benefits are greater in the left than in the right visual field and larger for the most crowded letter positions. Endogenous valid cues reduced errors due to confusing letter positions more than misidentifications, specifically for the most crowded letter positions. Therefore, shifting endogenous attention along a line of text is likely an important mechanism to alleviate the effects of crowding on encoding letters within words. Our results help set the premise for constructing theories about how specific mechanisms of attention support reading development in children. Understanding the link between reading development and attention mechanisms has far-reaching implications for effectively addressing the needs of children with reading disabilities.

## Introduction

Reading is a complex visual task that demands shifting and focusing spatial attention to extract information about the letters that compose words. As you read these lines, you are moving your eyes from word to word quite effortlessly, even though your visual field is cluttered with hundreds of letters. Selecting letters that form the current word and prioritizing the next word in the sentence are necessary steps to read fluidly. Indeed, due to a bottleneck in the word recognition circuitry^1^, multiple words compete with each other, and focal attention is required to select one word at a time^2,3^. The ability to identify letters and extract their relative positions within each word is also fundamental for word recognition^4^. This is particularly true for children learning to read. While some studies have shown associations between differences in selective attention and reading^5–7^, the existing literature has yet to demonstrate how various mechanisms of attention affect the way letter combinations are processed. In this study, we asked, how do two types of covert visual-spatial attention affect recognition of letters within a string?

In the study of visual perception, spatial attention is defined as a collection of processes that prioritize task-relevant locations in the visual field and filter out irrelevant information. Spatial attention can be deployed overtly, by moving our eyes to a specific location, or covertly, without eye movements. Covert spatial attention helps monitor the environment and to plan subsequent eye movements. Covert spatial attention (henceforth simply referred to as “attention”) can be directed to a certain location in space voluntarily, based on current goals, or involuntarily, due to the sudden onset of a peripheral stimulus. The former type is endogenous attention, and the latter is exogenous. It has been shown that these types of attention improve task performance in a variety of visual tasks including contrast sensitivity^8,9^, texture segmentation^10^, visual search^11^, and many others (for review see^12^). Despite the vast literature, it is difficult to extend our understanding of the effects of attention on processing low-level stimuli (e.g., Gabor patches) to highly specialized and complex visual stimuli like letter strings and tasks like multiple letter processing that are implicated in reading.

Both endogenous and exogenous cues have been shown to improve performance for targets appearing at cued locations relative to targets at uncued locations, for many different stimuli and tasks^13–17^. However, the specific perceptual effects of endogenous and exogenous attention differ in key ways: (1) the time course of exogenous and endogenous cueing effects are reported to differ^18,19^; (2) the magnitude of endogenous, but not exogenous, effects scale with the likelihood that relevant stimuli appear at the cued locations; (3) endogenous attention flexibly optimizes performance as a function of task demands^20–23^, whereas exogenous cues cannot be ignored even when known to be uninformative^24–26^; and (4) there are qualitatively different effects reported with exogenous and endogenous cues. For example, in a texture segmentation task, exogenous attention improves performance at the periphery (where spatial resolution is low) but impairs performance at the center (where resolution is already high), whereas endogenous attention always improves perception by flexibly adjusting to spatial resolution^23,27,28^. These differential effects limit us from extending and generalizing the effects of exogenous and endogenous attention from one context to another, especially, if the core question is in understanding the basic role of selection mechanisms involved in processing letter combinations. Therefore, by combining the multi-element processing task and manipulating spatial attention with pre-cues, we developed a paradigm relevant to reading that allows for each type of covert spatial attention to be independently manipulated.

Word recognition is limited by the ability to identify its component letters^4^. To test the ability to identify letters within a string, we used the multi-element processing task^29^. First introduced by Sperling^30^, whole and partial report versions of the multi-element processing tasks are measures of information extraction from brief visual displays. In the whole report version, the task is to report the identity of as many letters as possible from a briefly displayed string. In the partial report, the task is to report one of the letters at a location that is cued after the stimulus disappears. In our task, the elements are letters (all consonants) that do not form real words. This ensures that each letter must be accurately encoded independently. Some studies on crowding have used similar paradigms to understand how pre-cues influence the critical spacing between letters^31^. Other studies have used pre-cues and post-cues to understand mechanistic differences in letter identification between fluent and struggling readers^32,33^. A major difference between these studies and the current study lies in the fundamental question: our goal is to understand how endogenous and exogenous visual spatial attention affect multi-letter encoding. The previous studies by Castet and colleagues highlight the importance of considering iconic memory in dyslexia, but do not report how spatial pre-cues specifically affect performance on this task^33^. Unlike those used in crowding studies, our task does not manipulate the spacing between characters, and stimuli are presented in the central (as opposed to peripheral) visual field, which is most relevant for word recognition.

This task is also commonly cited in dyslexia literature. Some studies have reported that individuals with a sub-type of dyslexia exhibit deficits in both whole and partial report versions of the task^34–36^, and that this deficit might be causally related to reading development^34,37^. The reason for such a deficit is still not fully understood; some authors attribute deficits in the multi-letter processing task to the “visual attention span”, but the specific mechanism of attention that may or may not be involved has yet to be specified. Nevertheless, the multi-letter processing task reliably correlates with reading ability indicating the relevance of this measure for reading^35,38^. Here, we manipulate spatial pre-cues to specifically characterize the effects of exogenous and endogenous attention on simultaneously encoding multiple letters.

## Materials and methods

### Participants

We recruited 32 healthy adult participants (24 females, 8 males) between 18 and 35 years of age. Sample size was targeted to be twice that is typically targeted in visual attention studies that involve typical adult participants. All participants had normal or corrected-to-normal vision and gave written informed consent in accordance with the Stanford University Institutional Review Board. In addition, since the data collection was done during the pandemic, every measure was taken to comply with relevant guidelines at the time and to ensure safety of participants and experimenters. All experimental protocols were approved by the Stanford University Institutional Review Board. All participants had no known history of dyslexia, auditory deficits, or attention deficits. Each participant also completed a battery of standard reading assessments (TOWRE test Torgesen, J., et al. 1999). Participants had a TOWRE composite score at or above the population average score of [range: 100 to 129; 50th to 97th percentile] and TOWRE Phonemic Decoding Efficiency score [range 100 to 132, 50th to 98th percentile].

On each trial the participant is presented with a briefly flashed string of 6 letters, three to the left and three to the right of fixation. Each letter spanned 0.5^o^ of visual angle with 0.58° center-to-center spacing at 1.16 × the letter width. The eccentricities of the inner, middle, and outer letters on each side were 0.58°, 1.16°, and 1.74°, respectively. According to Bouma’s Law^39^, stimuli within the critical spacing (a distance less than half the eccentricity of the target) are known to cause crowding. Immediately after the letters disappear, a “post-cue” appears: a blue line under one of the letter locations. The participant’s task is to report the identity of the target letter that had been at the post-cue location. They do so by clicking on one of 12 letters presented below the post-cue (not shown). Spatial attention is manipulated before the letters appear by “pre-cues” that direct attention to either the left three letters or to the right three letters. Exogenous pre-cues are three red lines that flash briefly below the letter locations. They are uninformative, meaning that their locations are random with respect to the post-cued target’s location. Endogenous pre-cues are salient red lines presented adjacent to the center on one of the horizontal arms of the fixation cross (or on both horizontal arms of the fixation cross). They are informative, meaning that they indicate the side where the target will be. Therefore, endogenous pre-cues manipulate the voluntary deployment of attention towards task-relevant information, while exogenous pre-cues manipulate the involuntary capture of attention by salient stimuli. In all cases, participants are required to maintain central fixation (monitored by an eye tracker). For both types of cues, there is a “neutral” condition in which both sides are pre-cued, and the target could be at either side. A “valid” pre-cue is on the same side as the upcoming post-cue. An “invalid” pre-cue is on the opposite side of the post-cue. The effect of attention is measured via “cue effects”: the difference in target recognition accuracy between valid, neutral, and invalid trials. Note that the pre-cues are represented with the target strings in the figure to show the spacing between the letters and the pre-cue. Pre-cues appear before the target string in every trial sequence.

### Equipment and stimuli

We generated stimuli using MATLAB (The Mathworks Corporation, Natick, MA, USA) and the Psychophysics Toolbox^40,41^ on Linux (Ubuntu 16.04). We used the ViewPixx EEG display (1920 × 1080 resolution, 120 Hz refresh rate) that subtended 36 degrees of visual angle (36°) horizontally. Participants were comfortably seated at a head and chinrest 73 cm from the monitor and used the mouse to select their response from the screen. The screen background was set to white (100 cd/m^2^) and a black (4.3 cd/m^2^) fixation cross (0.5^o^x0.5°) was always present at the center of the screen. The target stimuli were a string of six letters, three to the left of the fixation cross and three to the right of the fixation cross. For each trial, six consonants were randomly sampled without replacement from a set of 12 letters [B, F, H, K, L, N, P, T, V, X, Y, Z], that match on perimetric complexity, which is defined for each letter as the sum of inside and outside perimeters of the foreground, squared, divided by the foreground area, divided by 4π^32^. All letters were in monospaced Dejavu font at 100% contrast. Letter height was set to 0.5° and the center-to-center letter spacing was 0.58°. Each six-letter string subtended 3.98°.

### Trial sequence

**Figure 1:**
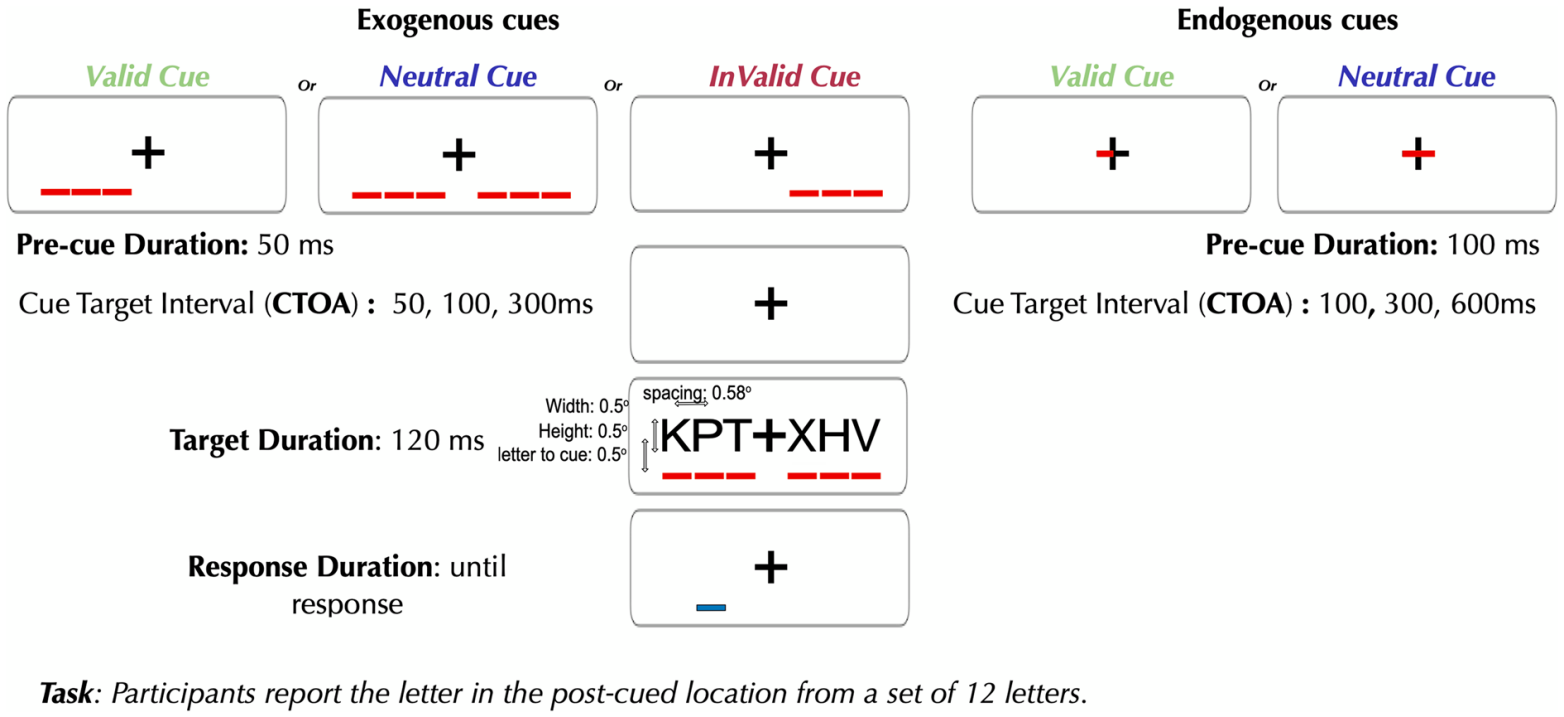
Example trial sequence for the exogenous and endogenous attention experiments in the letter recognition task.

An illustration of trial sequence with an exogenous and endogenous spatial cue is presented in Fig. 1. On each trial, after the pre-cue (described below), the participant is presented with a string of 6 letters flashed for 120 ms, three to the left and three to the right of fixation. Immediately after the letters disappear, a “post-cue” appears: a blue line under one of the letter locations. The participant’s task is to report the identity of the target letter that was at the post-cue location, by clicking on one letter from a set of 12 letter choices provided. The 12 letter choices comprise of all the 6 letters (including 1 target) presented in the string along with 6 letters that were not part of that trial’s string. Spatial attention was manipulated before the letters appear by “pre-cues” that direct attention to either the left three letters or to the right three letters.

### Pre-cues

To elicit exogenous shifts of spatial attention to either the left or right side, the cues were red lines flashed for 50 ms under the three letter positions before the onset of the string. The exogenous cues were uninformative as to the side of the target letter to be post-cued. To elicit endogenous shifts of spatial attention to the left or right side, the cue was a salient red line presented adjacent to the center on one of the horizontal arms of the fixation cross, indicating with 100% certainty the side of the target letter to be post-cued. Participants were explicitly instructed about the uninformative and informative nature of the cue in the exogenous and endogenous conditions, respectively. The cue conditions were run on separate days. The exogenous cue condition was always run before the endogenous condition. All cue conditions and CTOAs were randomized within each session and participants were allowed to take breaks at any point. Upon resuming after a break, participants were recalibrated to enable eye tracking for fixation breaks.

In all cases, participants were required to maintain central fixation (see eye tracking methods below). For both types of cues, there was a “neutral” condition in which both sides are pre-cued, and the target could be at either side. A “valid” pre-cue is on the same side as the upcoming post-cue. An “invalid” pre-cue (which only applies for exogenous) appears on the opposite side of the post-cue. In the exogenous cue condition, there is equal probability of a cue being valid, invalid or neutral, whereas half the endogenous cues were neutral, and the rest were all valid.

### Cue-target onset asynchrony (CTOA)

The time interval between the onset of a pre-cue and onset of the stimulus string is the cue-target onset asynchrony abbreviated as CTOA. We used CTOAs of 50, 100 and 300 ms for the exogenous cues and CTOAs of 100, 300 and 600 ms for the endogenous cues. These choices allowed us to measure the dynamics of the two cueing effects within their appropriate range. The reflexive, transient, exogenous system is known to operate at shorter intervals than the sustained, voluntary, endogenous system^18,19,42^.

There were 10 trials for each position (6 positions), for each of the CTOAs (3 CTOAs) and for each cue validity condition (3 for exogenous and 2 for endogenous cue conditions) totalling to 540 trials in the exogenous and 360 trials in the endogenous cue condition.

### Eye tracking

Eye position was monitored using the Eyelink 1000 plus (SR Research, Ontario, Canada). Any horizontal saccades greater than the 0.5° during the presentation of the letter string aborted the trial, which was added back to the end of the block. Any fixation outside of the screen center + /− 0.5° horizontally and 2° vertically pauses the experiment for a recalibration. Observers made fixation breaks on less than 5% of trials. Less than 1% of the eye gaze positions, across all participants, were outside of the central fixation window (1 deg) during the period from the onset of the cue to the offset of the target for both the exogenous and endogenous cue conditions. Thus, results are not due to eye movements towards the cued sides.

### Attentional effects

The effect of attention is measured as the difference in target recognition accuracy between valid, neutral and invalid trials. Based on extensive research on characterizing exogenous and endogenous attention^12^ we expect task performance with valid cues to be greater than that with neutral cues (and invalid, in the case of exogenous cue). The cue benefit is the difference in target recognition sensitivity between valid and neutral cue trials. For exogenous pre-cues, the cue cost is the cost of directing attention to the wrong side: the difference in sensitivity between neutral and invalid cue trials. Finally, the total cue effect, for the exogenous system, is the difference in sensitivity between valid and invalid cues.

### Inclusion criteria

All participants had normal or corrected normal vision, with no known history of dyslexia or attention disorders. Not all participants were native English speakers but all had standardized English reading scores that were average or above average (range: 100–132).

### Data analysis

Of the 32 subjects recruited, 4 were excluded from all the analysis: one who had ADHD, and three who could not return for the second visit. We used the Palamedes function PAL_SDT_MAFC_PCtoDP^43^, which converts proportion correct into d' for a standard M-alternative-forced-choice task, assuming an unbiased observer. For accuracy at floor (0%) we assume that had we run twice as many trials, 1 trial would be correct, so accuracy = 1/(2*Number of trials). When at ceiling (100%), we assume that had we run twice as many trials, there would be 1 incorrect trial, so accuracy = 1−(1/(2*Number of trials)).

We used linear mixed effects (LME) analyses and post-hoc t-tests to analyze accuracy across different CTOAs, letter positions, and cue validity conditions. The random effects consist of subject-dependent random intercepts and slopes. We used a maximal random effects structure (random intercepts and slopes^44^), in all our LME models unless specified. For example: the first LME model to the accuracy data (d’) with fixed effects of cue validity and CTOA and a maximal random effects structure, referred to in the Results section, is written as follows in the MATLAB Statistics toolbox or with the lme4^45^ package in R: d’ = cue_validity + CTOA + (cue_validity + CTOA | subject).

#### Reliability

For each participant, for each cue validity condition, we computed d’ separately on odd and even trials. The correlation across subjects between those split-half d’ levels indicates reliability. A Spearman-Brown correction was applied to the obtained correlation to adjust for the fact that only half the trials were used. The Spearman-Brown split-half reliabilities for d’ in the exogenous and endogenous neutral cue conditions were r = 0.91 and 0.89, respectively. For valid trials, reliabilities for the exogenous and endogenous cues were r = 0.91 and r = 0.87, respectively.

## Results

### Exogenous and endogenous spatial cues affect sensitivity in a multi-letter processing task

**Figure 2:**
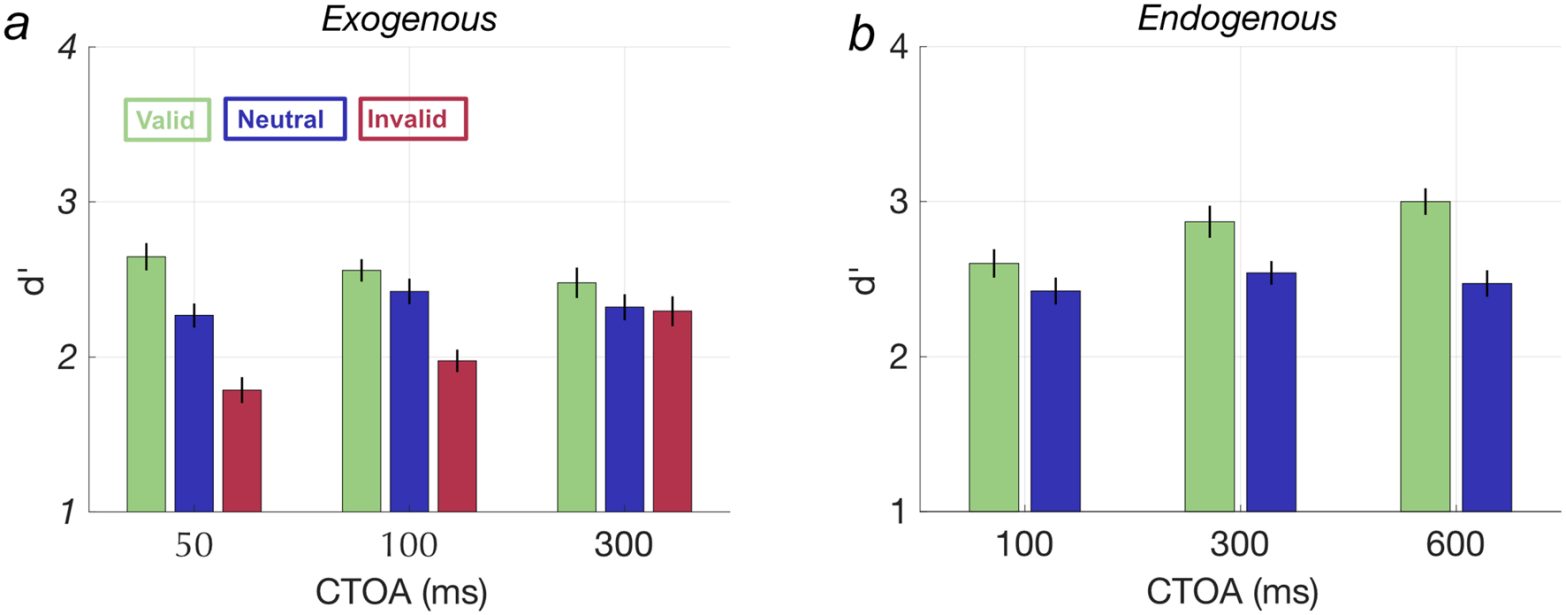
shows accuracy in letter recognition in the multi-letter processing task as a function of cue-target asynchrony. (**a**) results from the exogenous cue type and (**b**) results from endogenous cue type, across different cue validity trials (colors) and CTOAs. Error bars represent SEM.

We first established that exogenous and endogenous cues affect accuracy for identifying a single post-cued letter when participants are briefly presented with a horizontal string of 6 random letters centered on the fovea. Figure 2 shows accuracy (d’) averaged across all participants (n = 28) for each cue validity condition and CTOA for both exogenous (Fig. 2a) and endogenous (Fig. 2b) cue types. In general, the partial report task was difficult, and accuracy was consistently below 80% correct [Exogenous (M + /−SEM %): 73.03 ± 1.98; Endogenous: 76.60 ± 2.02]. Overall mean performance (d’) in the neutral cue condition was 2.33 (SE: 0.07). Notably, performance in the neutral cue trials (blue bars in Fig. 2) remained consistent across different CTOAs for both cue types [effect of CTOA for exogenous: F(2,81) = 0.9876; *p* = 0.38, and endogenous F(2,81) = 0.5209; *p* = 0.59], indicating that dividing attention is not affected by the interval between the cue and the target.

To examine the effect of exogenous spatial cues on task performance we fit a LME model to the accuracy data (d’) with fixed effects of cue validity and CTOA and a maximal random effects structure. d’ in the valid condition was greater than in the neutral condition, which was greater than in the invalid condition. Valid exogenous cues (when the pre-cued side matched the post-cued side) improved performance relative to neutral exogenous cues by, on average, 5.41% accuracy (d’ difference of 0.22; t(247) = 5.35, *p* = 1.94 × 10^–07^]. Invalid exogenous cues (when the pre-cued side was opposite to that of the post-cued side) decreased performance compared to neutral cues, on average, by 9.38% accuracy (d’ difference of 0.32; t(247) = − 7.93, *p* = 7.76 × 10^–14^]. Sensitivity also varied across CTOAs [F(2, 247) = 5.92, p = 0.0031]. Posthoc t-tests revealed that the CTOA of 50 ms was significantly higher from 100 ms [t(247) = 2.21, *p* = 0.028] and 300 ms [t(247) = 3.38, *p* = 0.00084].

In the endogenous condition, valid cues increased performance by, on average, 6.98% accuracy (d’ difference 0.31) compared to neutral cues [F(1,164) = 69.18; *p* = 3.29 × 10^–14^]. Sensitivity also varied across CTOAs [F(2,164) = 15.25; *p* = 8.44 × 10^–7^]; post hoc t-tests showed that performance differed between all three CTOAs [100:300 ms t(164) = − 5.15; *p* = 7.19 × 10^–7^; 100:600 ms t(128) = − 8.31; *p* = 3.29 × 10^–14^], with higher d’ for valid cues at the longest CTOA.

### Exogenous and endogenous attention exhibit different temporal dynamics

**Figure 3:**
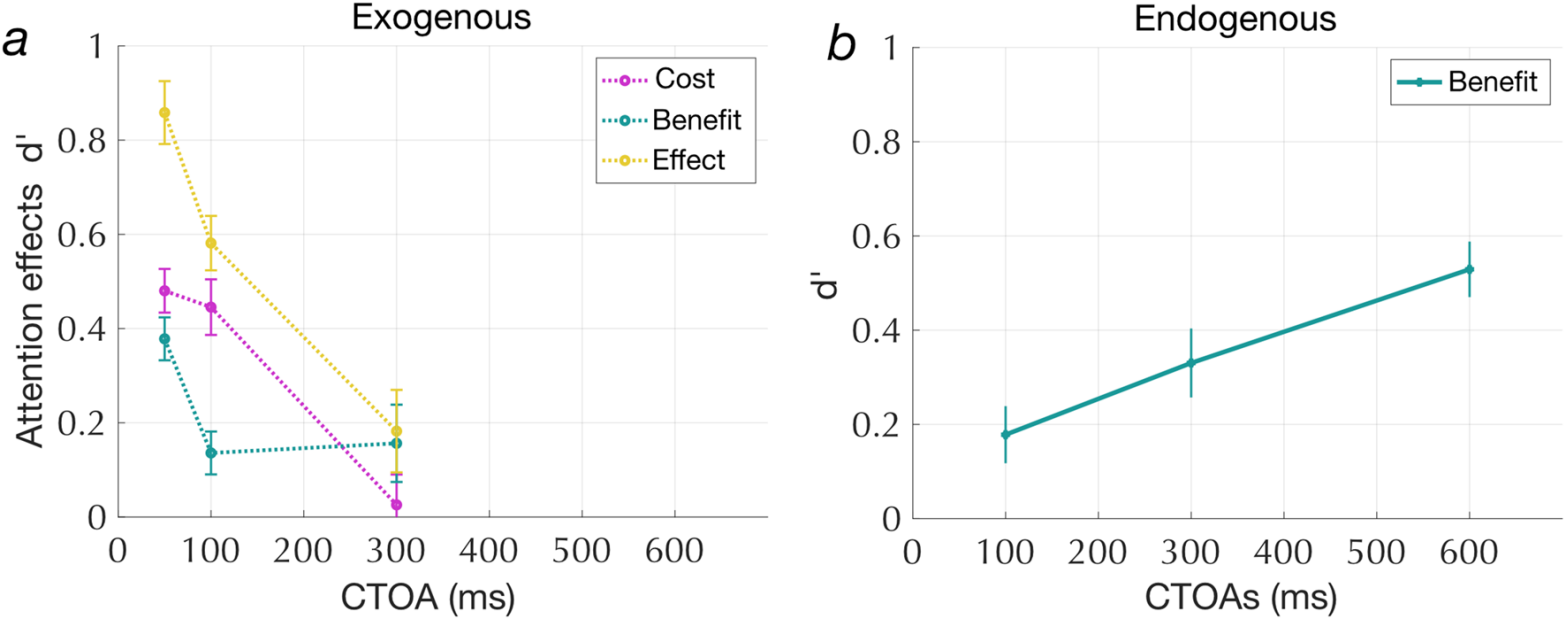
(**a**) Time course of cue benefits, cue cost and cue effects with exogenous cues CTOAs: 50, 100, 300 ms); (**b**) Endogenous cue benefits as a function of CTOAs (100, 300, 600 ms). Error bars represent SEM.

Based on prior studies, we expect the exogenous attention system to respond more quickly (within ~ 100 ms) to cues compared to the endogenous deployment of attention (~ 300 to 500 ms)^12^. The interval between the onset of the cue and the onset of the target is typically considered to be the interval during which attentional resources are allocated to the cued side^46^. To evaluate how cue benefits (valid - nuetral d’) change over time, we fit an LME model to cue benefits with CTOAs as fixed effects for each cue type. Figure 3 shows cue benefits for both exogenous (Fig. 3a) and endogenous cues (Fig. 3b) as a function of CTOA. Exogenous cue benefits were greatest at the shortest time interval and decayed at longer intervals [F(2,81) = 14.1, *p* = 5.57 × 10^–06^; post hoc t-tests: 50 ms > 100 ms: t(81) = 3.99; *p* = 1.04 × 10^–04^ and 100 > 300 ms: t(81) = 2.95; *p* = 0.004]. Endogenous cue benefits, on the other hand, increased as a function of CTOA, with maximum effect at the longest interval [F(2,81) = 8.88; *p* = 3.24 × 10^–04^; post hoc t-tests: 600 ms > 100 ms: t(81) = 4.11, *p* = 6.46 × 10^–05^ and 300 ms > 100 ms: t(81) = 2.31, *p* = 0.023].

Like the exogenous cue benefit, the exogenous cue cost (neutral–invalid) also has a time course (also see Supplementary). Figure 3a shows exogenous cue benefit, cue cost and total cue effect as a function of CTOA. A LME model was fit to cue cost with fixed effects of CTOA. Cue cost significantly varied as a function of CTOA [F(2,81) = 17.61; *p* = 4.46 × 10^–07^], being greatest at 50 ms, and declining thereafter [300 < 50 ms: t(81) = 5.55; *p* = 3.48 × 10^–7^; post hoc t-tests: 300 < 100 ms: t(81) = 5.23, *p* = 1.27 × 10^–06^]. The cue cost at a CTOA of 100 ms was not significantly different from that at 50 ms (t(81) = − 0.52; *p* = 0.60). Both exogenous benefits and costs were lower at a CTOA of 300 ms, suggesting that the effects decay with time for exogenous attention.

The following sections focus on the time points when each attention system has its maximal effects: 50 ms for exogenous cues and 600 ms for endogenous cues. Note that the endogenous cue benefits at 600 ms were slightly higher than exogenous cue benefits at 50 ms (Mean d’ difference = : 0.151; SE: 0.07; t(27) = 2.12; *p* = 0.041).

### Cue benefits interact with letter position within a string

**Figure 4:**
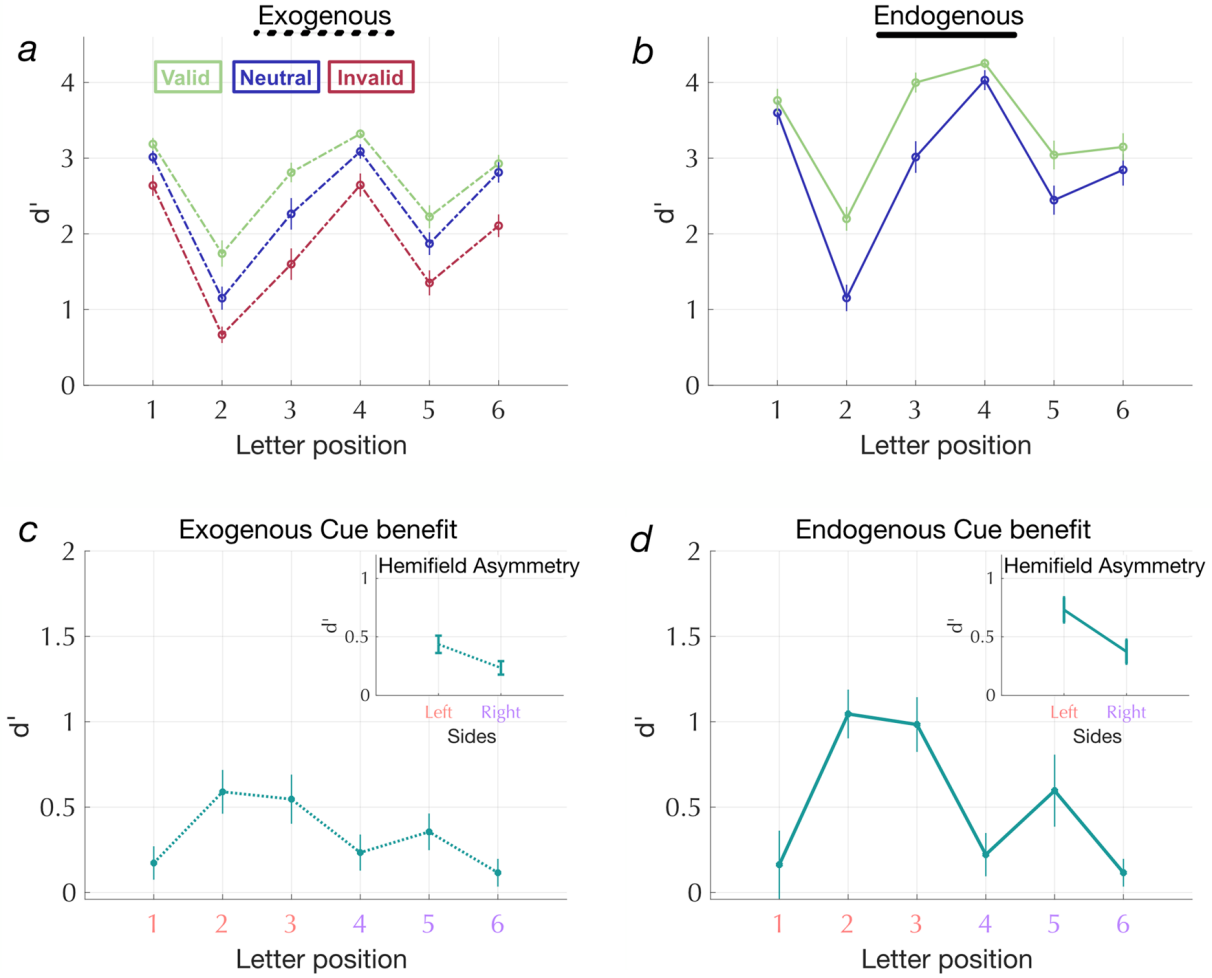
(**a**, **b**) Serial position functions are W-shaped for both cue types. (**c**, **d**) Cue-benefit functions for both cue types. Both exogenous and endogenous cue benefits show M-shaped profiles with larger cueing effects at crowded letter positions.

Many studies have shown that letter recognition accuracy differs across positions within a string^32,47,48^. In general, accuracy as a function of position follows a W-shape, with greater accuracy for outer positions and at fovea, and diminished accuracy for internal letters^32,48^. Consistent with the literature, we also see a W-shaped serial position function for letters in all conditions and cue types (see Fig. 4a and b for exogenous cues at 50 ms and endogenous cues at 600 ms, and Supplementary Fig. S1 and S2 show the overlapping CTOAs). The W-profile for letter recognition is thought to be due to a combination of various underlying mechanisms ranging from the drop in acuity with visual eccentricity, visual crowding and working memory^32,47^. Interestingly, we noticed that letter recognition was significantly better in the right compared to the left hemifield, replicating well-known hemifield asymmetries for word and letter recognition^39,49–52^. The asymmetry was present for both exogenous and endogenous cues [Neutral cue condition: significant effect of cue type F(1,332) = 6.87; *p* = 0.009 and hemifield F(1,322) = 16.01; *p* = 7.74 × 10^–5^].

Here we ask a novel question: does the serial position function interact with spatial attention? Specifically, do the effects of exogenous and endogenous pre-cues differ across letter positions in a string? To examine these questions, we first plot exogenous and endogenous cue benefits (valid-neutral) as a function of the post-cued letter position: we call this the cue benefit function (again computed with CTOAs 50 and 600 ms, respectively). If a valid cue simply increases letter recognition accuracy equally across all letter positions, then we would expect the cue benefit function to be flat. On the one hand, if attention further enhances letters in positions that already show high sensitivity (i.e., positions 1, 4, and 6 which have high sensitivity) then the cue benefit function would also be W-shaped. If, on the other hand, letters in crowded positions (i.e., in the troughs of the W-profiles) benefit more with an attentional cue, then the cue benefit function would be M-shaped.

Figures 4c and d show the cue benefit functions. We fit a LME model to cue benefits with both cue type and positions as fixed effects. We observed a significant main effect of position [F(5,324) = 11.27; *p* = 4.94 × 10^–10^] and cue type [F(1,324) = 6.40, *p* = 0.01] but no significant interaction between cue type and positions [F(5,324) = 1.49; *p* = 0.19]. A post-hoc t-test (with a Bonferroni adjusted p-threshold for multiple comparisons set to: p_Adjusted_ = 0.010) comparing benefits at each position revealed that cue benefits only at position 1 was significantly lower than the average cue benefits across all other positions [t_1_(324) = − 3.42; p_1_ = 7.1 × 10^–4^]. Cue benefits at positions 2 and 3 were significantly higher than the average across all other positions [t_2_(324) = 4.04, p_2_ = 6.53 × 10–5; t_3_(324) = 3.17, p_3_ = 0.002]. Thus the M-shaped cue benefit function shows that the serial position function interacts with spatial cues and exogenous and endogenous attention both enhance sensitivity to crowded letter positions. Next, we asked how cue benefits vary between hemifields. Specifically, how does attention interact with the right hemifield bias for encoding letter strings?

### Letters are easier to recognize in the right visual field, but cue benefits are larger in the left

**Figure 5:**
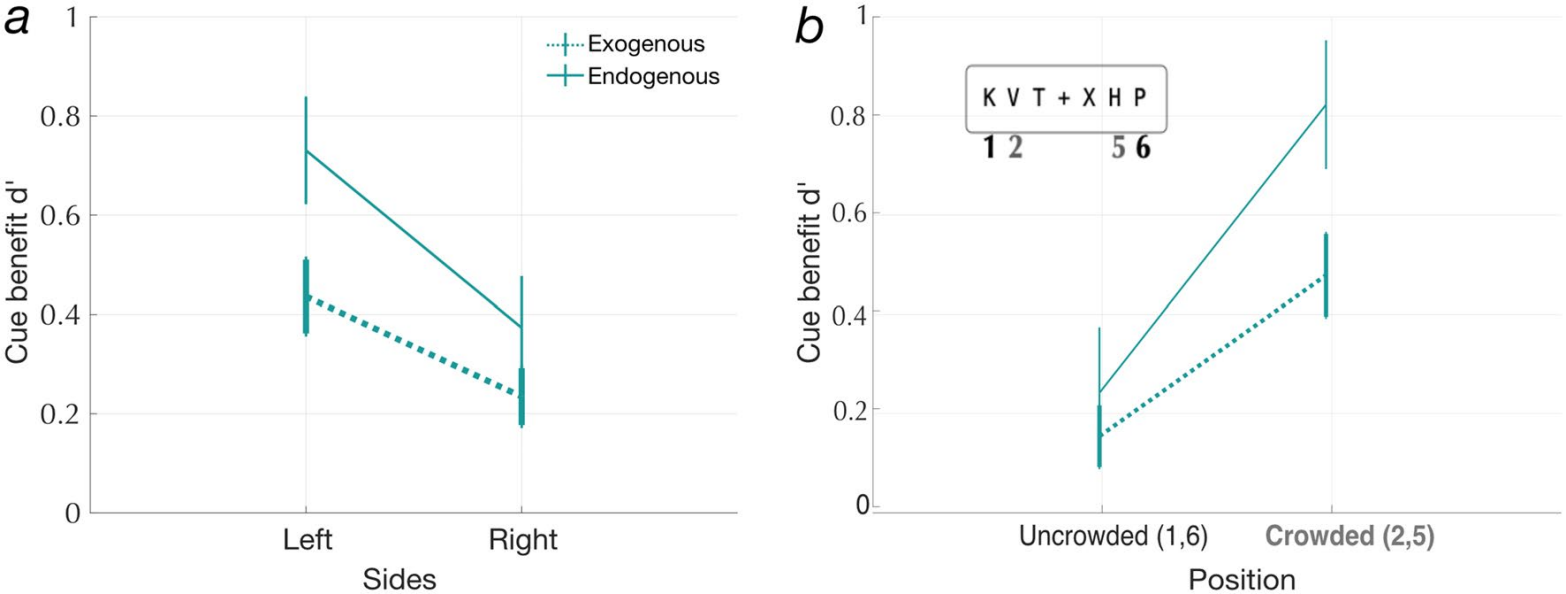
(**a**) cue benefits for the left and right hemifield. We see a significant main effect of cue type and hemifield but no significant interaction effect. (**b**) cue benefit for the most crowded and the least crowded letter positions. We see a significant main effect of letter crowding but no significant effect of cue type and no interaction between cue type and letter crowding.

We observe a significant hemifield asymmetry with both cue types (see inset plots in Fig. 4c, d and Fig. 5a). Fitting a LME on cue benefits with cue type and positions as fixed effects showed that cue benefits were significantly larger for the letter positions in the left hemifield (positions: 1, 2, and 3) compared to the right hemifield (positions: 4, 5 and 6) [F(1,332) = 10.17; *p* = 0.0016]. As previously reported, cue benefits were larger overall for endogenous than exogenous cues [F(1, 332) = 5.41;  *p*  = 0.021]. However the interaction between cue type and hemifield was not significant [F(1,332) = 0.87; *p* =  0.35]. Thus, spatial attention effects are greater in the left hemifield, where letter recognition accuracy is lower.

### Cue benefits are larger for letters in the most crowded positions within the string

A letter that can be easily identified when presented alone becomes unrecognizable when presented alongside nearby flankers, a phenomenon called visual crowding^39,53,54^. To compare cue benefits at the most crowded and least crowded positions, we took the outermost letters at positions 1 and 6 and compared them to the crowded letter positions 2 and 5. Figure 5b shows that cue benefits for the crowded letter positions (2 and 5) were higher than the outermost letters (1 and 6). A LME model was fit to cue benefits with cue type (exogenous or endogenous) and letter crowding (crowded or uncrowded) as fixed effects. There was a significant main effect of letter crowding [F(1,220) = 15.88; *p* = 9.165 × 10^–5^], but not of cue type [F(1,220) = 2.83; *p* = 0.09] nor a significant interaction [F(1,220) = 1.45; *p* = 0.23] (Fig. 5b). To verify that the difference in cue benefits across the two positions was not due to differences in baseline performance in the task, we split our participants into low performance (< 90% correct; N = 10) and high performance (> = 90% correct; N = 18) groups based on task accuracy in the neutral condition specifically at letter position 1. We see that the effect of letter crowding on the cue benefit (difference between benefits at the crowded and uncrowded letter positions) is significant for both groups [low neutral performance: F(1,76) = 5.83; *p* = 0.018; high neutral performance: F(1,132) = 8.83; *p* = 0.003]. Therefore,we see the same effect even in participants who are not close to the ceiling. Thus, both exogenous and endogenous cues benefit letters in the crowded positions more than those in the uncrowded positions.

### How do spatial cues reduce errors?

**Figure 6:**
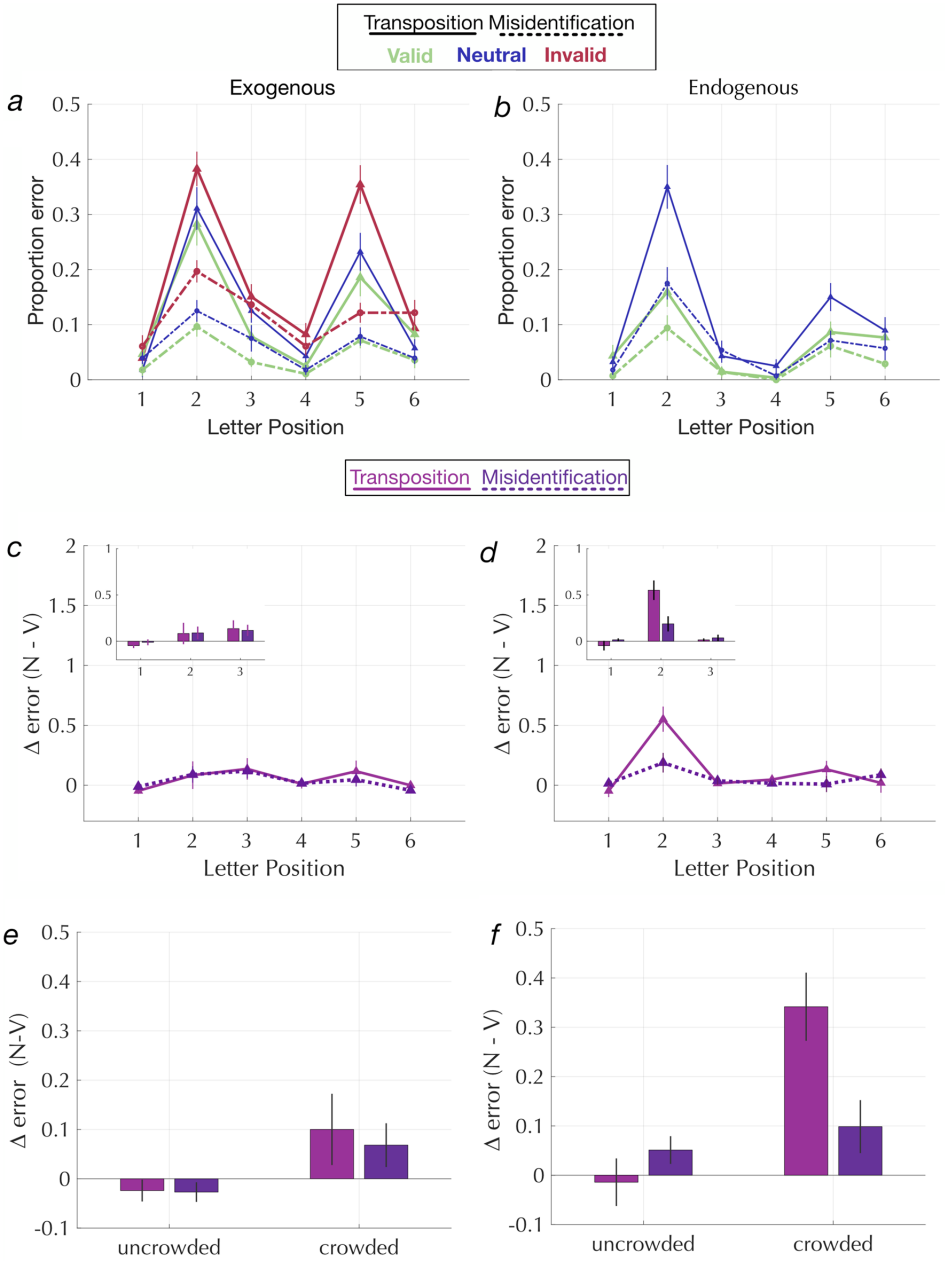
(left: Exogenous and right: endogenous). (**a**, **b**) show error profiles, proportion transposition and misidentification errors as a function of letter position for trial types with neutral cues (blue lines), valid cues (green lines) and invalid cues (red lines). Transposition errors are ~ 2 × more likely than misidentification errors overall. (**c**, **d**): show the linearized difference in errors with a neutral vs. valid cue. Exogenous valid cues reduce transposition errors uniformly across all letter positions, however endogenous valid cues decrease both transposition and misidentification errors in the crowded positions more compared to uncrowded positions. The inset figures show the same information as bar graphs for the three letter positions in the left hemifield. It is clear that endogenous valid cues reduce transposition errors for the most crowded position in the left hemifield. (**e**, **f**) Errors calculated for letter positions 1 and 6 (uncrowded) and positions 2 and 5 (crowded). We show that endogenous valid cues reduce transposition errors particularly for those letters in the crowded positions.

To understand how exogenous and endogenous cues increase letter recognition accuracy within a string, we evaluated the types of errors made on incorrect trials. A transposition error is when the participant confuses the relative positions and reports an adjacent letter in the string instead of the target letter. For example, in a target string “K N B T Y P”, reporting T or P would be classified as a transposition error if the post-cued target was Y. A misidentification error is when the participant reports a letter that was not present in the string. For example, in the same target string, a misidentification error would be reporting V instead of the target Y. We computed the proportions of both error types as the number of errors divided by the total number of trials, separately for each position and cue condition. The resulting error profile is shown in Fig. 6a and b. Note that the error profile is the inverse of the W-shaped profile for accuracy. Error rates are higher at each position with neutral cues (blue curves) than with valid cues (green curves). With invalid exogenous cues (red curves), errors are higher compared to neutral cues. This difference in error rates with a valid compared to a neutral cue is expressed as the difference in proportion errors between the two conditions (N–V).

We then assessed the difference in these error rates between valid and neutral cue conditions to better understand how valid cues improve overall accuracy. Since differences in proportions are non-linear, we first linearize the proportion error data by putting them through the inverse of the cumulative normal distribution function. We then defined ∆ error as the difference between those normalized error rates in the neutral - valid conditions (see norminv for calculations, Fig. 6c and d).

We then fit a LME model to ∆ error for each cue type with letter position and error type as fixed effects. With exogenous cues, ∆ errors showed a significant effect of letter position [F(5,324) = 4.71; *p* = 3.6 × 10^–4^] but no significant effect of error type [F(1, 324) = 0.12; *p* = 0.73] or interaction between error type and letter position [F(5,324) = 0.44; *p* = 0.82]. A post hoc t-test (with a Bonferroni adjusted p-threshold for multiple comparisons: p_Adjusted_ = 0.010) showed that ∆ errors at position 1 were significantly lower compared to the average ∆ errors across all other positions (t_1_(324) = − 2.81; *p* = 0.005). It should be noted that ∆ errors at the first letter position are close to zero because valid cues don’t benefit recognition much at position 1. With endogenous cues, ∆ errors showed a significant effect of letter position [F(5,324) = 7.89; *p* = 4.94 × 10^–7^], no significant effect of error type [F(1,324) = 2.72 ; *p* = 0.09], but a significant interaction between letter position and error type [F(5,324) = 2.87; *p* = 0.015]. A post hoc t-test showed that transposition errors at position 2 were significantly higher compared to errors at all other positions (t_2_(324) = 2.55; *p* = 0.01). Thus a larger cue benefit in the left hemifield with both endogenous and exogenous valid cues (shown in Fig. 4c and d) is due to a reduction in both transposition and misidentification errors. Interestingly, only with endogenous valid cues we observe a significant interaction between error type and letter position showing that valid endogenous cues reduce transposition errors for the most crowded letter position in the hemifield with reduced sensitivity (position 2).

To understand how the two cue benefits interact with letter crowding (shown in Fig. 5b), we analyzed how the cues affect the proportion of misidentification and transposition errors for crowded positions (2 and 5) and uncrowded positions (1 and 6). A three-way mixed effects model was fit to the ∆errors with cue type (exogenous or endogenous); error type (transposition or misidentification) and letter crowding (crowded or uncrowded) as fixed effects and a full random structure. There was a significant main effect of letter crowding [F(1,440) = 20.56; *p* = 0.00001], with greater ∆error for the crowded than uncrowded positions. There was a significant main effect of cue type [F(1440) = 6.875; *p* = 0.009], with larger reductions in errors with valid endogenous than exogenous cues, and a significant three way interaction between letter crowding, error type and cue type [F(1440) = 4.148; *p* = 0.038] showing that the interaction between letter crowding and error type [F(1440) = 6.034; *p* = 0.012] varies with the type of cue (see Fig. 6e and f). A post hoc comparison showed that transposition errors were significantly reduced in the crowded letter positions with endogenous valid cues [t(98.35) = 4.223; bonferroni adjusted p value for multiple comparison *p* = 0.0015]. Note that there is no two-way interaction between letter crowding and error type for exogenous cues. Thus, although both exogenous and endogenous cues benefit the crowded letter positions more than the uncrowded letter positions, endogenous valid cues reduce transposition errors to a greater extent than exogenous valid cues for crowded letters (seen in Fig. 6f).

## Discussion

We compared the effects of exogenous and endogenous spatial attention on performance in the multi-letter processing task. Our results show that both exogenous and endogenous attention affect letter recognition, increasing accuracy at the cued location and decreasing accuracy at the uncued locations. Consistent with previous studies^12,25,55^, we observe that exogenous and endogenous attention operate at different time scales (Fig. 3). Exogenous cue benefits were greatest at the shortest cue-to-target interval (50 ms) and then declined, whereas endogenous cue benefits were greatest at the longest cue-to-target interval (600 ms).

For both cue types, valid cues improved accuracy the most for those letter positions where accuracy was the lowest in the neutral cue condition. Specifically, cue benefits were higher for the left hemifield (similar to findings reported in a recent study^52^) and for the middle (positions 2 and 5) letter positions. Endogenous cue benefits were generally larger than exogenous cue benefits (note that valid endogenous pre-cues indicated the target side with 100% spatial certainty). Valid endogenous cues were especially effective at reducing transposition errors for the most crowded letters in the left visual field. In other words, our results suggest that endogenous attention (volitional control of attentional resources) helps in correctly encoding letter order within a string, a skill fundamental to word recognition.

Our results also align with studies on crowding that have shown that attracting spatial attention by precuing the target location diminishes crowding^31,56–58^. We show that valid spatial cues benefit the crowded letter positions more than the uncrowded letter positions (Fig. 5b) for both exogenous and endogenous spatial cues. Our task and paradigm was not designed to measure critical spacing (distance between target and adjacent letters). However, we still might speculate on the mechanisms by which attention improves performance. For example, the difference in transposition errors between endogenous and exogenous cues suggests that there are multiple mechanisms by which attention improves performance. Specifically, we might speculate from our results that the specific effect of endogenous attention on transposition errors might reflect a reduction in critical spacing. Nevertheless, this is a hypothesis that requires further follow up with experiments that manipulate spacing in the multi-letter processing task.

Further many authors purport that visual attention plays a causal role in dyslexia (the most prevalent learning disability). However many studies do not isolate the role of specific attentional mechanisms, and many do not clearly operationalize “attention”^5,6,34–37,59^. Others who have operationalized attention show correlations with cueing effects but with tasks that have no clear relevance to reading^5–7,60^. Some such paradigms that don’t clearly isolate exogenous and endogenous attentional mechanisms^7^. Our data are the first to compare the effects of endogenous and exogenous spatial cues, in terms of letter identity and letter order perception and time course.

The processing of text—specifically letters within a word (string)—is affected by many inherent limitations of the visual system that results in the W-shaped position function for letter recognition within a string. For example, internal letters are more difficult to identify due to crowding, but there are other mechanisms at play as well^32,33,47,61,62^. Whatever the underlying mechanisms are that result in the W-shaped profile, those factors can be ameliorated by voluntary focusing of spatial attention which ultimately helps encode letter order. It is interesting that we found these effects even though the spatial cues were not directed to specific letter positions, but rather to the left or right side of fixation. Therefore, voluntarily focusing attention to a subset of letters helps to correctly recognize their order.

Endogenous attention is likely to be particularly important for learning to read because it enhances information encoding at letter positions where sensitivity is the lowest. It is a feature of the endogenous attentional system to flexibly adapt to task-specific demands, as has been reported in other tasks like texture segmentation^23,63^.

## Conclusion

Our results are the first to isolate the role of different spatial attentional mechanisms in letter encoding, a process fundamental to word recognition. We have systematically characterized the effect of two distinct selection processes in a task relevant for reading, and our results demonstrate some similar and some distinct effects on how readers encode the identity and position of letters within a string. This work lays the foundation for two important future lines of research: (1) evaluating specific attention mechanisms in reading disability, based on concrete hypotheses. For example, based on our findings we can now hypothesize that endogenous attention is critical for reading, particularly in young children learning to read; and (2) tracking the developmental trajectory of attentional mechanisms to further understand how development shapes attention and intersects with the development of reading ability.

## Supplementary Information

Supplementary Information 1.

Supplementary Information 2.

Supplementary Information 3.

Supplementary Information 4.

## Data Availability

Data supporting this work is available at the project’s Git repository [https://github.com/yeatmanlab/SpatialAttention_LetterEncoding.git].

## Code availability

Code to reproduce figures and analyses is available at the project’s Git repository [https://github.com/yeatmanlab/SpatialAttention_LetterEncoding.git].
