# Supplementary figures and images for: Spatial attention in encoding letter combinations
Source: Sci Rep. 2021 Dec 17;11:24179. doi: 10.1038/s41598-021-03558-4 (PMC8683492; doi:10.1038/s41598-021-03558-4)

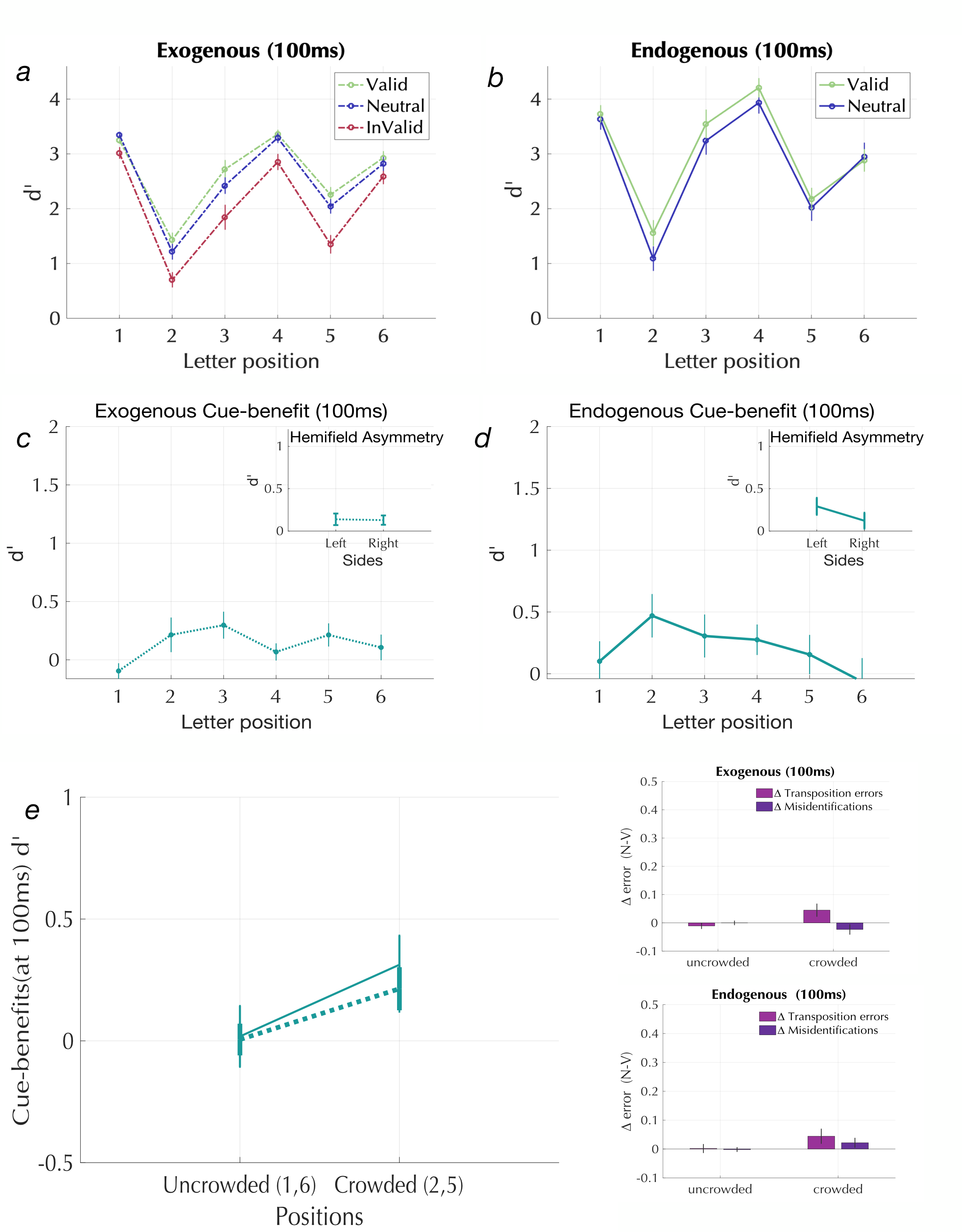

Supplement: Supplementary file 1 — Supplementary Information 1. [file 41598_2021_3558_MOESM1_ESM.tif]

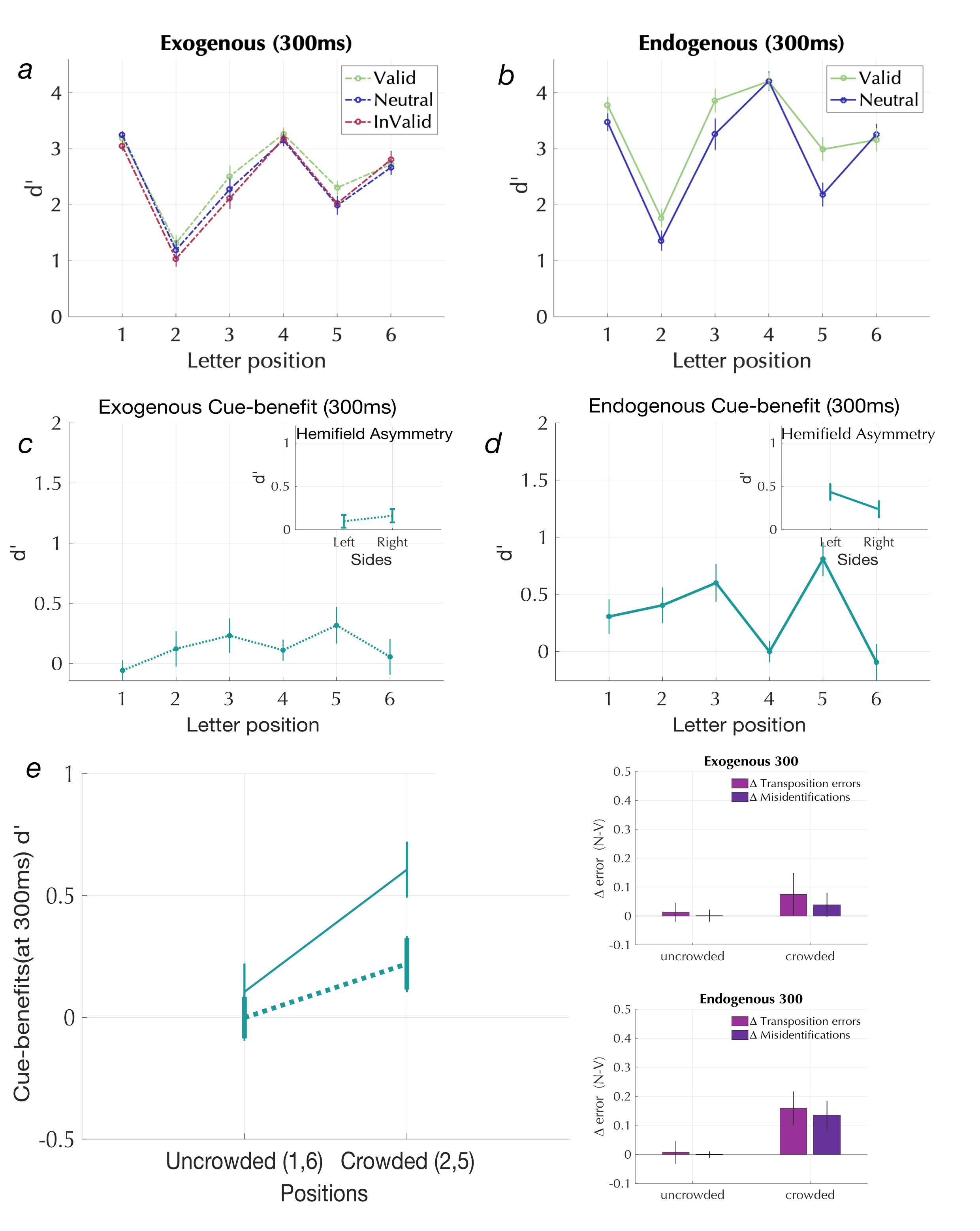

Supplement: Supplementary file 2 — Supplementary Information 2. [file 41598_2021_3558_MOESM2_ESM.tif]

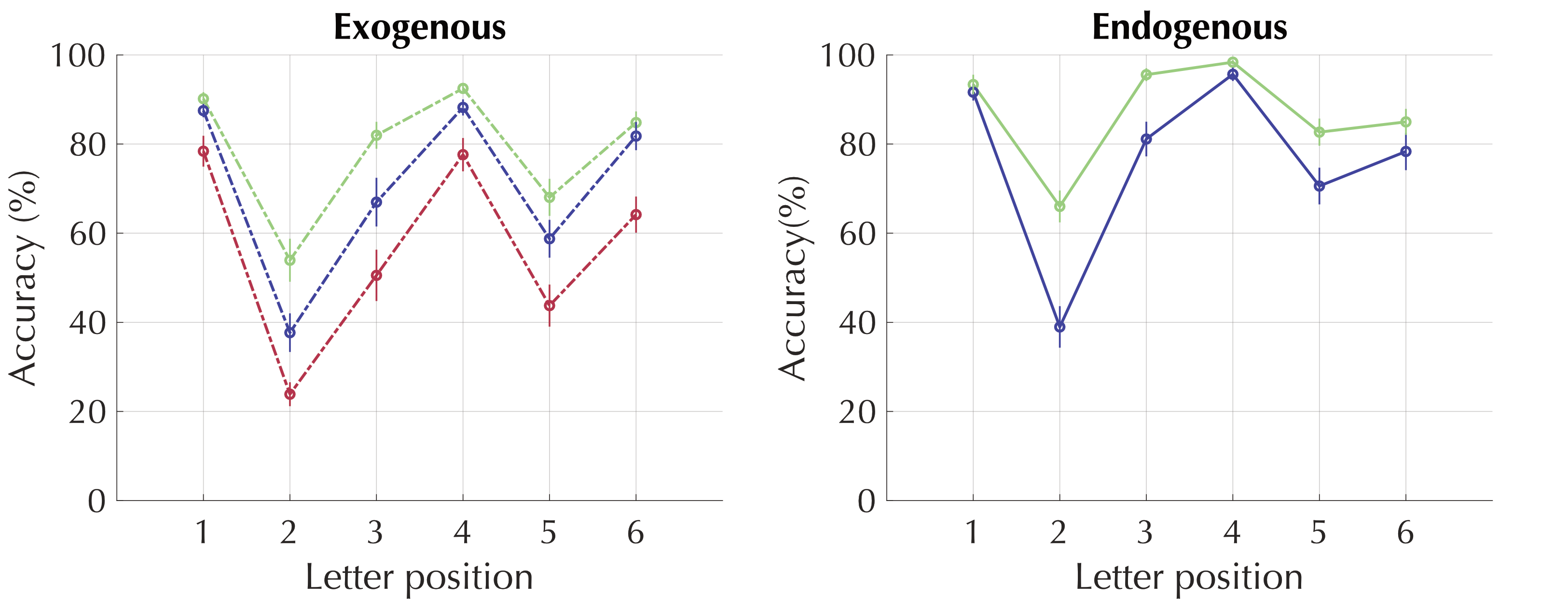

Supplement: Supplementary file 3 — Supplementary Information 3. [file 41598_2021_3558_MOESM3_ESM.png]
