# Supplementary material for: Spatial attention in encoding letter combinations
Source: Sci Rep. 2021 Dec 17;11:24179. doi: 10.1038/s41598-021-03558-4 (PMC8683492; doi:10.1038/s41598-021-03558-4)
Supplement: Supplementary file 4 — Supplementary Information 4. [file 41598_2021_3558_MOESM4_ESM.docx]

Supplementary:

***Different time course for Exogenous cue benefits and cue costs.*** We observed different time courses for the cue benefit, and cue cost effects as a function of CTOA with the exogenous cues (see Fig.3a). An LME on cue benefits [F(2,81)=14.1, p=5.57x10^-6^] and cue cost [F(2,81)=17.61, p=4.45x10^-7^] with CTOAs as fixed effects and a full random structure showed a significant main effect of CTOAs. A post hoc t-test comparing the effects at each of the CTOAs showed that cue benefits at 50 ms was greater than at 100 (t(81)= 3.99; p=1.04x10^-04^). However, interestingly, cue cost at a CTOA of 100ms was not significantly different from that at 50ms (t(81)= -0.52; p=0.60). It has been shown that with more time between the cue and the target, endogenous attention allows the observer to reorient and suppress exogenous distractors [^61^](https://paperpile.com/c/mBi5o9/GMQbx). Therefore, the drop in cue cost and cue benefit at a CTOA of 300 ms could reflect mitigating effects due to endogenous control. But the differences in time course for cue benefit and cue cost was surprising and we don’t know what might explain these differences. Previous studies using orientation discrimination and spatial acutiy tasks [^26,62,63^](https://paperpile.com/c/mBi5o9/Zaae+C6hN+xDU9) have reported differences in the magnitude of cue cost and cue benefits but the exact source of these effects are inconclusive.

***Exogenous and endogenous effects at CTOAs 100 and 300ms.*** Figure S1 shows the W-profiles, serial position function of cue benefits and error profiles for the 100 and 300ms CTOA, respectively. Our results bring to question the CTOA interval choice used in spatial cueing paradigms. This is critical when asking what attentional mechanisms are necessary and sufficient for normal reading development. Since the current study focused only on skilled adult readers, it is beyond the scope of the current paper to answer developmental questions. Future work is needed to understand how these attention systems develop, whether the time-course of attentional deployment changes, and how they may be related to reading proficiency. For instance: does development optimize the time it takes to respond to exogenous and endogenous cues? And are developmental differences reflected in the magnitude or time course of attentional effects? These questions are at the heart of theories linking reading development and attention [^56,64^](https://paperpile.com/c/mBi5o9/OuW7+5y8i).

***
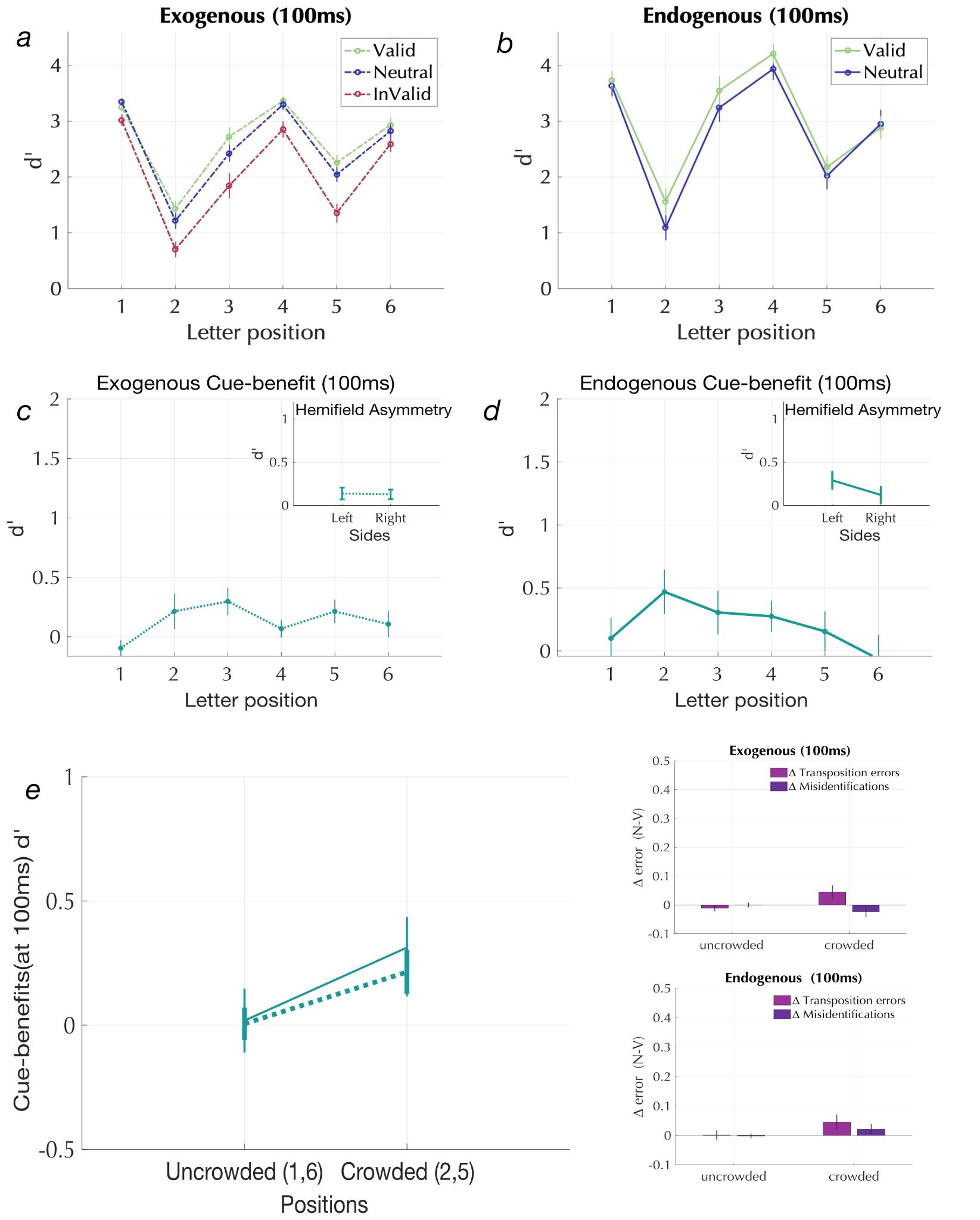
***

***Fig. S1: W-profile, cue benefit function, hemifield asymmetry and errors from exogenous and endogenous cues at a cue to target interval of 100ms.***


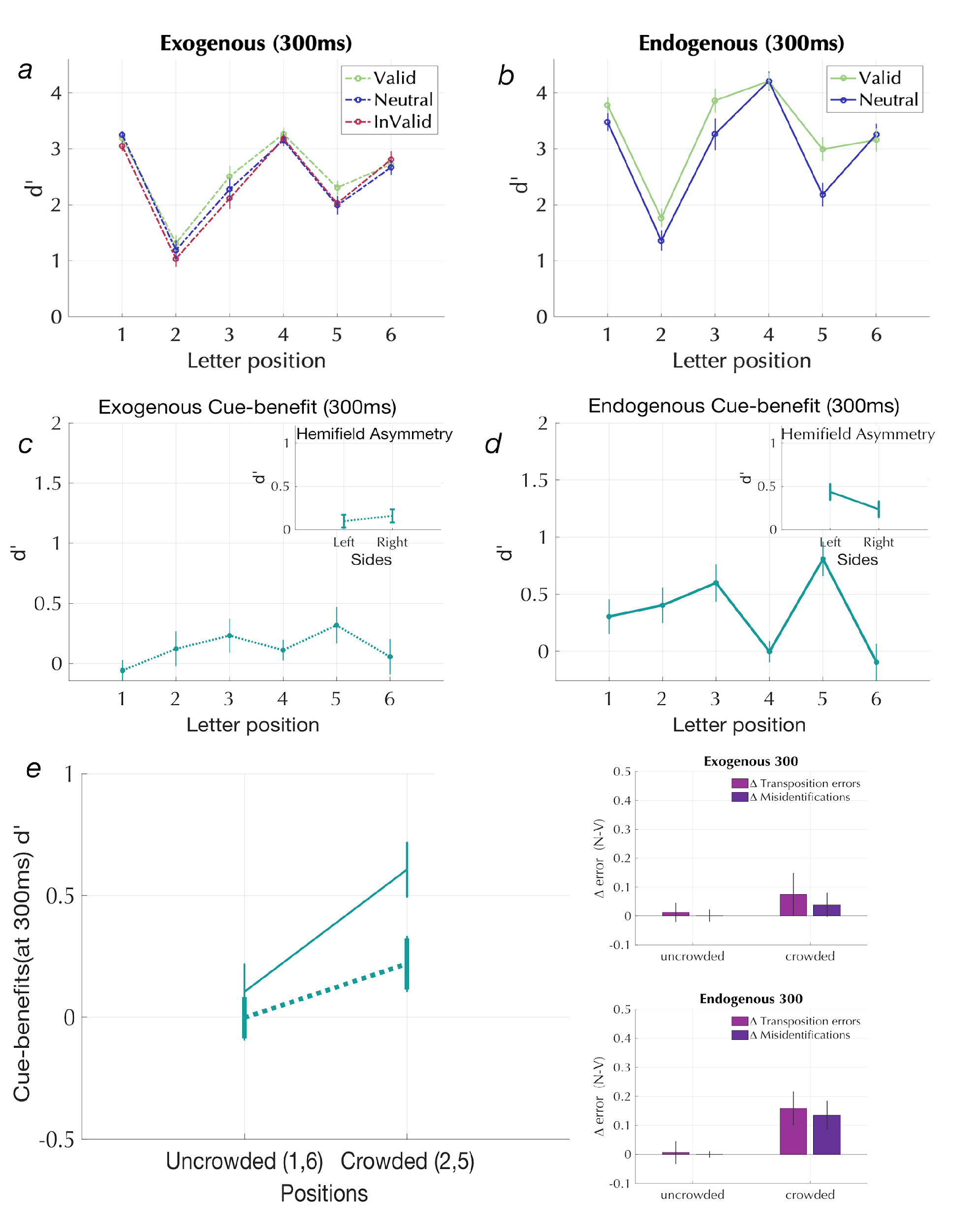


***Fig. S2: W-profile, cue benefit function, hemifield asymmetry and errors from exogenous and endogenous cues at a cue to target interval of 300ms.***

***W-shaped serial position function in percent correct:*** In figure 4 a and b we show the w-shaped serial position function (d’ as a function of different letter positions). The accuracy equivalent of Figure 4 a and 4b is presented here.

*
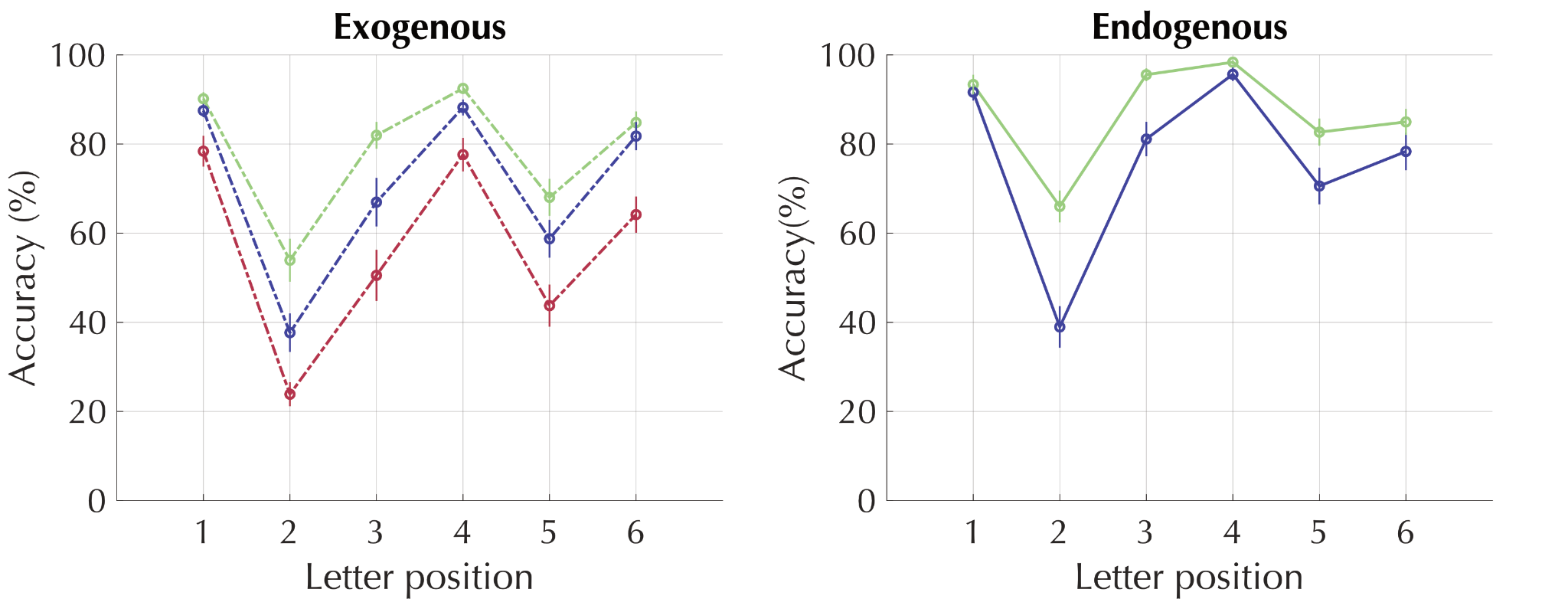
*

***Fig. S3. Accuracy as a function of letter position. The figure is equivalent to that presented in Figure 4 with percent correct in the y-axis.***
